# Strengthening Mentorship in Global Health for US Medical Students

**DOI:** 10.5334/aogh.4106

**Published:** 2023-09-27

**Authors:** Olamide Jarrett, Linda Humaidan-Zayed, Stacey Chamberlain, Stevan Weine

**Affiliations:** 1 Academic Programs, UIC Center for Global Health, US; 2 Section of Infectious Diseases, UIC Department of Medicine, US; 3 UIC College of Medicine, Center for Global Health (CGH), US; 4 UIC Department of Emergency Medicine, Director of Academic Programs, UIC Center for Global Health, US; 5 Department of Psychiatry, Director of Global Medicine & Director of the Center for Global Health, US

**Keywords:** mentorship, global health, medical, academia

## Abstract

US medical students demonstrate strong interest in receiving global health training. In 2012, the Center for Global Health (CGH) at the University of Illinois College of Medicine (UICOM) developed a Global Medicine (GMED) program to match this interest. From its initiation, mentorship has been a key component of the GMED program. More recently, this has been strengthened by applying additional evidence-informed approaches toward mentoring. These include the “mentor up” approach, a “network of mentors,” and an individualized development plan (IDP). Applying these changes were associated with increases in the number of student abstract presentations and peer-reviewed journal publications. Mentorship based upon evidence-informed approaches should be a key component of global health education in academic medical centers.

## Background

Over the past several decades, there has been a strong interest in global health among US medical students, and many medical school programs are meeting these interests by developing global health training opportunities [1,2]. In 2019, the Association of American Medical Colleges conducted a survey which found that almost a quarter of graduating medical students participated in a global health experience, emphasizing the need for promoting global health competencies in medical school training [3,4]. Although there is a lack of consensus around which global health competencies should be implemented within a medical global health training program, the importance of global health training through mentorship has gained widespread attention as university-based global health programs have proliferated, and the number of students engaging in global health has risen [4,5]. For example, a multi-institution Medical Student Global Health Study Group found that 65% of US fourth-year students in the survey envisioned practicing internationally in some capacity [6].

Students have cited mentorship as an important component of their global health training, guiding students in contributing to sustainability, health equity, and cultural competency in population-healthcare [4,7,8]. According to the National Academies of Sciences, Engineering, and Medicine, “Mentorship is a professional, working alliance in which individuals work together over time to support the personal and professional growth, development, and success of the relational partners through the provision of career and psychosocial support [9].” Multiple studies of medical education have shown that mentoring is associated with increased career guidance and satisfaction, personal and professional development, specialty and academic career choice, career retention, research guidance, access to resources, and research productivity [10,11,12,13]. Studies also found that those with exposure to mentoring have a greater belief in their ability to accomplish specific goals and tasks [13,14,15,16].

Although mentoring is important for aspiring clinicians, researchers, and educators, studies show that less than half of medical trainees actually have mentors [8]. Many US medical students lack adequate global health preparation and may have limited clinical or research experience in a global health setting which can also negatively impact their host community [1,17,18]. With many global health trainees spending time in low- and middle-income countries (LMICs), mentorship is also needed to help the mentee understand the appropriate attitudes, capacities, and roles in such settings and the many challenges they may face [18,19]. More global health mentors are needed both at US medical schools and in international settings, where mentees are learning new skills in unfamiliar environments [20].

## The Global Medicine Program

In 2012, the University of Illinois College of Medicine (UICOM) established the Global Medicine (GMED) program to address the interests of medical students in global health training. The GMED program is a longitudinal four-year track for select medical students (12 per class) and is housed within the Center for Global Health (CGH) at the UICOM. A core element of GMED is completing a global health capstone project over the course of the four-year program.

In 2019, new evidence-based strategies for strengthening mentorship of the GMED program were applied to better support the medical students in completing their global health capstone project. One strategy was implementing the “mentor up” approach [21,22]. “Mentor up” is a business approach applied to medical trainees where “the mentee takes ownership of and directs the relationship, letting the mentor know what they need and communicating the way their mentor prefers [22].” The Center for the Improvement of Mentored Experience in Research (CIMER) also uses the mentor up approach to aid early stage investigators to better address their needs through effective communications with their mentors [21]. Other global health training programs who applied this management approach found that the health management skills of fellow trainees were improved and aided in the implementation and completion of over 50% of proposed projects [23]. This evidence-informed approach appeared well-suited to help global health-focused medical trainees take ownership and accountability of their mentoring needs through a management perspective [21].

A “network of mentors” was another evidence-based strategy that was applied within the GMED program. One individual faculty member cannot fulfill all of the mentoring needs of a medical trainee, necessitating a “network of mentors [24,25].” A network introduces the student to mentors with varying skill sets and areas of expertise, provides a safeguard from inadequate mentoring, and shows recognition that as mentoring needs evolve, the composition of the network may also change [24,26,27]. In the GMED program, this required identifying and recruiting additional mentors and clarifying their roles.

The third evidence-based strategy was incorporating an individualized development plan (IDP) for each global health medical student. This concept emphasizes the importance of mentees individually developing a written plan that articulates their short- and medium-term goals [21]. Incorporating an IDP is part of managing mentor-mentee relationships, a component of the mentor up approach [21]. Applying an IDP helps medical students prioritize their needs and set goals to make themselves accountable for achieving these goals throughout the academic year. It also provides an opportunity for the students to learn how to better write and articulate their goals.

## Previous Challenges in the GMED Program

In 2012, the initial student-mentor structure consisted of four core faculty from the CGH leadership serving as academic mentors for all 48 GMED students. The role of the academic mentor was to help students set academic goals and provide career advice within the context of developing a global health career. In addition, the students each needed to identify and select a capstone project mentor with whom they shared research interests and who would guide the student in the completion of their capstone project. The four core faculty serving as academic mentors for 48 students were usually also serving as capstone project mentors for multiple students. This structure created several challenges to providing adequate mentorship to our GMED students.

One challenge was insufficient time to provide adequate mentoring for all the GMED students. Each of the four core CGH faculty served as an academic mentor to 12 students in addition to serving as research mentors for other students. This led to unrealistic time demands on faculty and sometimes meant that mentors were not always available when GMED students sought their mentorship. Thus, some projects were sometimes completed with no faculty guidance, which led to quality issues in the deliverance of students’ final capstone projects and presentations. Another challenge was the inconsistent mentoring of students throughout their capstone projects. In choosing their mentor, many students experienced strong mentor-mentee relationships. However, others experienced loss of mentors due to faculty leaving UICOM for other positions and no longer being able to supervise the student, causing a delay in student project completion and a lack of proper support. Table 1 illustrates some of the challenges students identified in their reflection papers regarding their GMED capstone completion during the initial mentorship program. (Note: The anonymous quotations contained in Tables 1 and 2 come from final student reflection papers done to facilitate quality improvement).

**Table 1:** Student challenges in prior GMED mentorship structure and evidence-based strategies used to improve the mentorship program.


STUDENT FEEDBACK ON CHALLENGES FACED DURING GMED CAPSTONE PROJECT COMPLETION IN PREVIOUS MENTORSHIP PROGRAM		EVIDENCED-BASED STRATEGY USED TO ADDRESS CHALLENGE

Trouble identifying a capstone project	“After two years of searching for a suitable capstone project, I was stumped. … As a lowly, lone medical student, I did not find much enthusiasm or support. After this setback, I was discouraged and briefly abandoned the idea of working with [population of interest].” “The Capstone was very difficult for me when I first entered medical school. I did not have a firm idea of what I wanted to work on.”	An assigned academic mentor assists the student in identifying a capstone project mentor and provides consistent mentorship throughout program.

Lack of experience in developing a research project	“The experience included many bumpy rides. Some of the reasons [include] poor experience in research design on my end.” “Running a validated method is still a bit of a struggle for me. I collected data three times, and each time things went slightly differently, making comparison of each set impossible.”	An individualized development plan (IDP) allows students to stay on track and receive better follow-up and guidance from mentors.

Lack of financial and human resources	“This quickly proved to be very difficult with significantly less manpower and funding available. This realization showed me that I had to tackle a smaller part of the problem at hand.” “I think at this point in my medical career, I was used to setbacks but my capstone project had changed multiple times (due to lack of mentors) and each time it became increasingly more difficult to keep going and reassess the situation and see where I can pick up the pieces, or to start completely anew.”	The mentor up approach teaches students to better vocalize their needs to their mentors and receive the support they need. A network of mentors provides greater access to human and other resources to benefit students.


**Table 2:** Student feedback of current GMED mentorship program 18 months after implementation of mentor program updates.


STUDENT FEEDBACK ON BENEFITS OF CURRENT GMED MENTORSHIP PROGRAM DURING CAPSTONE PROJECT COMPLETION	

Strengthened and improved research skills	“I now have a better understanding of the process it takes to conduct a research project and the patience and perseverance it takes to keep a project going.” “I had never previously been exposed to concepts like social determinants of health or generating a longitudinal relationship with community partners. While in hindsight they seem intuitive, without GMED I would never have considered incorporating them into future research projects and my practice.”

Expansion of diverse faculty mentorship	“I was extremely fortunate to have different mentors that were dedicated to helping me.” “I was happy to see multiple different fields represented by our faculty and their individual research projects. It is truly rare to have a program so willing to listen to their students and adjust the structure of curriculum to reflect the concerns that students have (specifically diversity and colonialism). GMED fostered an open environment where we could all share and learn from one another.”

Encouraged development of faculty relationships	“The greatest and most valuable thing I’ve gained through the GMED program is without a doubt the relationships I’ve formed with classmates and faculty members.” “The emphasis on mentorship and importance of regular check-ins instilled greater confidence in my work and encouraged me to work even harder to accomplish something truly worthy of publication. It also pushed me to develop strong relationships with my team.”


The above issues were further exacerbated by students not understanding how to manage their mentor relationships. Some students did not set regular times to meet with their mentors to discuss their projects. Others did not understand the best protocol for communicating with their mentors. For example, many students reported never sending a follow-up message to faculty after not receiving a response to an initial email. Depending on mentors to push forward with student projects not only causes a delay in completing projects, but also creates a lack of accountability from students. In 2019, the above challenges were addressed in strengthening the GMED mentorship program through: (1) educating students on how to best manage mentor/mentee relationships from the mentor up approach; (2) implementing a network of mentors by expanding the pool of faculty providing various expertise in academic mentoring to GMED students; and (3) ensuring students have consistent mentoring throughout their participation in GMED, even if their research projects and research mentors changed.

## Strategies for Strengthening the GMED Program

To ensure adequate academic mentoring for our students, the GMED program implemented the network of mentors approach, which expanded the pool of academic mentors to include all core CGH faculty and a larger number of newly recruited affiliate faculty. Expanding the mentor pool significantly reduced the number of students each faculty member advised to one to three GMED students per academic year. This led to faculty having more time to provide adequate support to each GMED student. Each student is still assigned an academic mentor and selects their own capstone project mentor, both of whom they meet with regularly over the course of their four-year curriculum. Academic mentors are assigned at the start of the GMED program, allowing students to receive mentorship from the beginning of their time in GMED. Additionally, having the same academic mentor over the course of four years allowed students to have one consistent mentor who was familiar with their progress over the course of their GMED experience. The program recommends that students connect with their assigned academic mentor quarterly, either in person or via email updates.

The students identify capstone project mentors via multiple avenues, including recommendations by academic mentors, speed networking events, or through prior relationships students have with selected capstone project mentors from previous global health projects. Those GMED students who are involved in LMIC-based projects also identify local partners and collaborators who function as mentors. These LMIC partners or collaborators meet individually with the students and may also join mentor committee meetings as described below. However, we do not burden these LMIC partners with all the institutional responsibilities which would come with them being UIC faculty, as we recognize our LMIC partners’ time constraints’ and faculty responsibilities at their home institutions.

The program requires biannual mentor committee meetings with the mentee and both mentors as a group to discuss overall student progress (both academic/career goals and research). During these meetings, the students create an IDP to set priorities that need to be completed throughout the term to reach their main goal. The IDP helps students take accountability for their academic goals while giving their mentors a clear picture of what the students need to work on throughout the term. Having a group meeting avoids confusion and inconsistency around specific GMED requirements for their capstone projects, especially when capstone project mentors are not affiliated with CGH. Students also benefit from “group thinking” when discussing their projects as the academic mentor can serve as an additional source of feedback regarding their research progress.

In implementing the mentor up approach, the GMED program created a mentoring guide for the students and for academic and capstone project mentors that clearly outlined their roles, as well as what they can expect from their respective mentor/mentee. For students, this included information on best communication practices with mentors, such as following up on emails when they do not hear back and creating their IDP. For mentors, this included tips regarding best practices, such as allowing students to develop their own ideas and not forcing their suggestions on students, which has been particularly beneficial to junior faculty with more limited mentoring experience. The guidebook has signature pages for mentors and mentees to attest that they have read the guidebook and understand mentoring expectations.

In addition to the mentoring guidebook, an information session is held at the start of the first year of medical school to explain the mentor system and assign academic mentors. Annual information sessions are also held with the core and affiliate GMED faculty to explain the mentoring system and mentoring responsibilities. In all cases, any faculty or student with questions or issues regarding the mentoring structure can contact the Associate Director of Academic Programs who oversees the mentoring program.

Lastly, there is a formal reporting system to keep track of students’ progress, in which students must submit a report after their biannual mentor committee meetings that outlines: (1) the overall aim of their project; (2) their goals for the previous six months; (3) the progress they made in accomplishing those goals; (4) barriers to accomplishing those goals; and (5) their goals for the next six months. Having this formal reporting system encourages students to stay on top of their mentor relationship by taking accountability. It also allows the faculty leadership to quickly identify students who are experiencing significant barriers in their progress and require additional assistance.

## Learned Lessons in Mentorship Strengthening

After 18 months of implementation, there have been several benefits noted by the GMED students regarding the implementation of the revised GMED mentoring program. In several cases, the program was able to intervene in a timely manner so that students were not left without a project in their fourth and final year. Many students have reported in their evaluations and reflection papers the benefits of the mentor up system and increased level of faculty involvement and support. The expectation that students mentor up helped many students develop an understanding of how to communicate with their mentors and increased their professionalism in managing relationships. It also helped them to stay accountable for their goals by developing their IDPs.

The COVID-19 pandemic had a major impact on students’ overall experience in medical school and especially in their global health experience (e.g., opportunities to travel and develop research projects in other countries). We noted a reluctance of some students to meet with mentors when they seemingly had no good project opportunities. We encouraged students to draw upon their mentors to problem solve, and many were able to pivot and complete quality projects despite not being able to travel to complete their projects. Table 2 illustrates the reflections given by the students’ experiences after strengthening mentorship elements in the GMED program, highlighting non-identifiable quotes.

**Figure 1:**
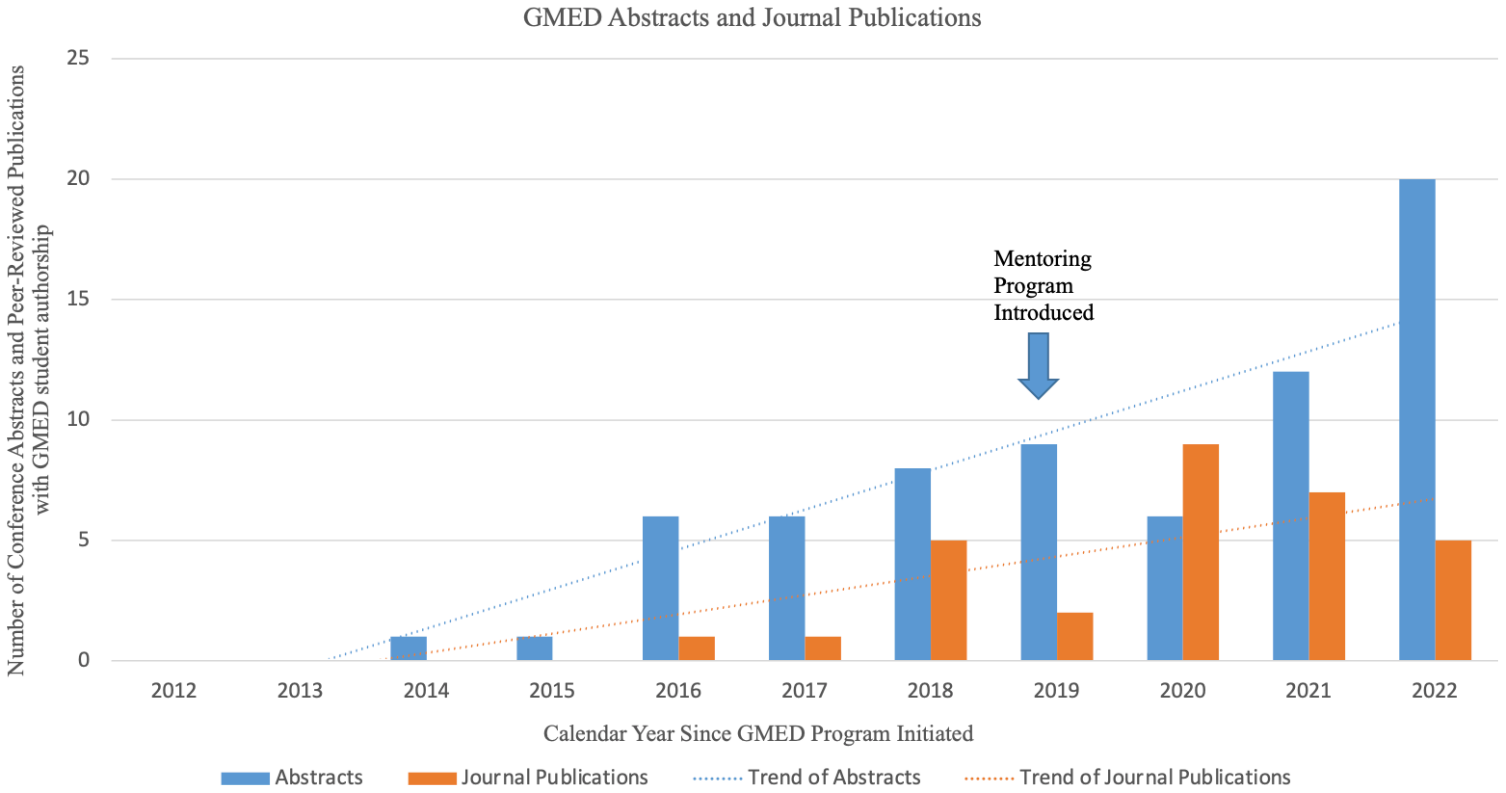
Trend in peer-reviewed abstracts and journal articles written by GMED students before and after changes to the GMED mentorship program.

The lessons learned in implementing and strengthening a global health program for medical students has led to improved productivity of students completing their projects on time and their projects leading to abstracts and/or publications. In the seven years from 2012 to 2019, GMED students presented 31 peer-reviewed abstracts and published 9 manuscripts. After strengthening the GMED mentorship program in 2019, there were 38 abstract presentations in just three years and 21 peer-reviewed journal publications (see Figure 1).

## Future Implications and Limitations

To further strengthen the GMED program, we plan to develop more comprehensive monitoring and evaluation of mentorship. Dimensions of mentorship that can be evaluated may include: (1) the mentor’s advising skills; (2) the mentee’s achievement of both knowledge and skills-based global health compentancies, and; (3) research and project outcomes. Overall research and project outcomes can be evaluated by continued monitoring of abstracts and publications. Mentorship skills can be evaluated through a Mentoring Competency Assessment (MCA) completed by each mentor and mentee [28]. Tracking additional metrics and outcomes (such as faculty feedback) may improve the rigor of the program, which is a current limitation of the mentorship program. Additionally, CGH seeks to identify other ways of formalizing the roles of LMIC mentors without unduly burdening them with institutional responsibilities.

Overall, the improved mentorship model has added value to our initial GMED program and may be useful to adapt in other academic training programs, broadening the model’s scalability.

## Conclusion

The initial experiences and lessons learned from implementing a mentorship model in the GMED program has shown how valuable mentorship is for global health medical students. Applying strategies such as the mentor up approach, facilitating a network of mentors, and incorporating an individualized development plan (IDP) has provided better support and guidance for students in their academic and research careers. Furthermore, the implementation of a mentorship model has trained students and faculty on how to manage their mentor-mentee relationships. With continued efforts, we plan to measure satisfaction and evaluation of mentorship among faculty and students as well as outcomes, such as the number of publications and continued pursuit of global health research careers, as more objective markers of the benefits of the mentorship program.

## References

[1] Nelson BD, Kasper J, Hibberd PL, Thea DM, Herlihy JM. Developing a career in global health: considerations for physicians-in-training and academic mentors. J Grad Med Educ. 2012; 4(3): 301–306. DOI: 10.4300/JGME-D-11-00299.1 23997872 PMC3444181

[2] Slifko SE, Vielot NA, Becker-Dreps S, et al. Students with global experiences during medical school are more likely to work in settings that focus on the underserved: an observational study from a public US institution. BMC Med Educ. 2021; 21: 552. DOI: 10.1186/s12909-021-02975-3 34715843 PMC8556999

[3] Association of American Medical Colleges (AAMC). Medical School Graduation Questionnaire. Association of American Medical Colleges; 2020. Available at: https://www.aamc.org/media/46851/download.

[4] Adams LV, Wagner CM, Nutt CT, et al. The future of global health education: training for equity in global health. BMC Med Educ. 2016; 16: 296. DOI: 10.1186/s12909-016-0820-0 27871276 PMC5117699

[5] Rodríguez DC, Jessani NS, Zunt J, Ardila-Gómez S, Muwanguzi PA, Atanga SN, Sunguya B, Farquhar C, Nasuuna E. Experiential learning and mentorship in global health leadership programs: capturing lessons from across the globe. Ann Glob Health. 2021; 87(1): 61. DOI: 10.5334/aogh.3194 34307064 PMC8284496

[6] Kaeppler C, Holmberg P, Tam RP, Porada K, Stryker SD, Conway K, Schubert C, Medical Student Global Health Study Group. The impact of global health opportunities on residency selection. BMC Med Educ. 2021; 21(1): 384. DOI: 10.1186/s12909-021-02795-5 34266446 PMC8280583

[7] Frei E, Stamm M, Buddeberg-Fischer B. Mentoring programs for medical students—a review of the PubMed literature 2000–2008. BMC Med Educ. 2010; 10: 32. DOI: 10.1186/1472-6920-10-32 20433727 PMC2881011

[8] Fallatah HI, Soo Park Y, Farsi J, Tekian A. Mentoring clinical-year medical students: factors contributing to effective mentoring. J Med Educ Curr Devel. 2018; 5: 1–6. DOI: 10.1177/2382120518757717 PMC5824905 29497707

[9] National Academies of Sciences, Engineering, and Medicine. The Science of Effective Mentorship in STEMM. The National Academies Press; 2019. 31958221

[10] Schrubbe KF. Mentorship: a critical component for professional growth and academic success. J Dent Educ. 2004; 68(3): 324–328. DOI: 10.1002/j.0022-0337.2004.68.3.tb03748.x 15038633

[11] Sambunjak D, Straus SE, Marušić A. Mentoring in academic medicine: a systematic review. J Am Med Assoc. 2006; 296(9): 1103–1115. DOI: 10.1001/jama.296.9.1103 16954490

[12] Ramanan RA, Taylor WC, Davis RB, Phillips RS. Mentoring matters: mentoring and career preparation in internal medicine residency training. J Gen Intern Med. 2006; 21(4): 340–345. DOI: 10.1111/j.1525-1497.2006.00346.x 16686809 PMC1484727

[13] Nimmons D, Giny S, Rosenthal J. Medical student mentoring programs: current insights. Adv Med Educ Pract. 2019; 10: 113–123. DOI: 10.2147/AMEP.S154974 30881173 PMC6404673

[14] Feldman MD, Steinauer JE, Khalili M, Huang L, Kahn JS, Lee KA, et al. A mentor development program for clinical translational science faculty leads to sustained, improved confidence in mentoring skills. Clin Transl Sci. 2012; 5(4): 362–367. DOI: 10.1111/j.1752-8062.2012.00419.x 22883616 PMC3582327

[15] Garman KA, Wingard DL, Reznik V. Development of junior faculty’s self-efficacy: outcomes of a national center of leadership in academic medicine. Acad Med. 2001; 76: S74–76. DOI: 10.1097/00001888-200110001-00025 11597879

[16] Allen TD, Eby LT, Lentz E. Mentorship behaviors and mentorship quality associated with formal programs: closing the gap between research and practice. J Appl Psychol. 2006; 91(3): 567–578. DOI: 10.1037/0021-9010.91.3.567 16737355

[17] Shah S, Wu T. The medical student global health experience: professionalism and ethical implications. J Med Ethics. 2008; 34(5): 375–378. DOI: 10.1136/jme.2006.019265 18448720

[18] Shah SK, Nodell B, Montano SM, Behrens C, Zunt JR. Clinical research, and global health: mentoring the next generation of health care students. Glob Public Health. 2011; 6(3): 234–246. DOI: 10.1080/17441692.2010.494248 20635270 PMC2958226

[19] Prasad S, Sopdie E, Meya D, Kalbarcyzk A, Garcia P. Conceptual framework of mentoring in low and middle- income countries to advance global health. Am J Trop Med Hyg. 2019; 100(1): 9–14. DOI: 10.4269/ajtmh.18-0557 PMC6329351 30430983

[20] Bennett S, Paina L, Ssengooba F, Waswa D, M’Imunya JM. Mentorship in African health research training programs: an exploratory study of Fogarty international center programs in Kenya and Uganda. Educ Health. 2013; 26(3): 183–187. DOI: 10.4103/1357-6283.126001 25001352

[21] Center for the Improvement of Mentored Experiences in Research (CIMER). Cimerproject.org. Managing Mentoring Relationships Curricula and Training: Mentoring Up. https://cimerproject.org/mentoring-up/. Accessed February 15, 2023.

[22] Zerzan JT, Hess R, Schur E, Phillips RS, Rigotti N. Making the most of mentors: a guide for mentees. Acad Med. 2009; 84(1): 140–144. DOI: 10.1097/ACM.0b013e3181906e8f 19116494

[23] Prado AM, Pearson AA, Bertelsen NS. Management training in global health education: a Health Innovation Fellowship training program to bring healthcare to low-income communities in Central America. Glob Health Action. 2018; 11(1): 1408359. DOI: 10.1080/16549716.2017.1408359 29320943 PMC7011984

[24] Higgins MC, Kram KE. Reconceptualizing mentoring at work: a developmental network perspective. Acad Manage Rev. 2001; 26(2): 264–88. DOI: 10.2307/259122

[25] De Janasz SC, Sullivan SE, Whiting V. Mentor networks and career success: lessons for turbulent times. Acad Manage Perspect. 2003 ; 17(4): 78–91. DOI: 10.5465/ame.2003.11851850

[26] DeCastro R, Sambuco D, Ubel PA, Stewart A, Jagsi R. Mentor networks in academic medicine: moving beyond a dyadic conception of mentoring for junior faculty researchers. J Assoc Am Med Coll. 2013; 88(4): 488–496. DOI: 10.1097/ACM.0b013e318285d302 PMC3610810 23425990

[27] De Janasz SC, Sullivan SE. Multiple mentoring in academe: developing the professional network. J Vocat Behav. 2004; 64(2): 263–283. DOI: 10.1016/j.jvb.2002.07.001

[28] Cole DC, Johnson N, Mejia R, et al. Mentoring health researchers globally: diverse experiences, programmes, challenges, and responses. Glob Public Health. 2016; 11(9): 1093–108. DOI: 10.1080/17441692.2015.1057091 26234691 PMC5020346

